# NEIGHBORHOOD DISADVANTAGE AND COGNITION AMONG AFRICAN AMERICANS IN THE BALTIMORE STUDY OF BLACK AGING

**DOI:** 10.1093/geroni/igac059.454

**Published:** 2022-12-20

**Authors:** Adrienne Aiken Morgan

**Affiliations:** University of North Carolina at Chapel Hill, Chapel Hill, North Carolina, United States

## Abstract

Previous research has suggested an important and understudied association between neighborhood disadvantage and cognitive functioning among older African Americans. Data from the Baltimore Study of Black Aging (N=602) was utilized to examine the associations between Area Deprivation Index (ADI) and cognition. Multiple stepwise regression models, controlling for age, sex, education, and income, showed participants who scored better on measures of immediate memory, vocabulary, reasoning, and everyday cognition had lower ADI (less disadvantaged neighborhoods; p < .05). Interestingly, individuals living with higher ADI had better attention/working memory scores and better self-reported memory (p < .05). ADI was significantly associated with improvements or declines in performance, depending on the cognitive ability (e.g., learning and memory, working memory, global mental status, and visuospatial skill, p < .05). These findings suggest the importance of accounting for neighborhood context in African Americans’ cognitive aging research.

